# Aqua­(1,10-phenanthroline)(pyridine-2,6-dicarboxyl­ato)nickel(II) pyridine-2,6-di­carboxylic acid solvate tetra­hydrate

**DOI:** 10.1107/S1600536808039378

**Published:** 2008-12-03

**Authors:** Javad Safaei-Ghomi, Hossein Aghabozorg, Elham Motyeian, Mohammad Ghadermazi

**Affiliations:** a Department of Chemistry, Faculty of Science, University of Kashan, 51167 Kashan, Iran; b Faculty of Chemistry, Tarbiat Moallem University, Tehran, Iran; c Department of Chemistry, Faculty of Science, Payame Noor University (PNU), Qom, Iran; d Department of Chemistry, University of Kurdistan, Sanandaj, Iran

## Abstract

The title compound, [Ni(C_7_H_3_NO_4_)(C_12_H_8_N_2_)(H_2_O)]·C_7_H_5_NO_4_·4H_2_O or [Ni(pydc)(phen)(H_2_O)].pydcH_2_·4H_2_O, was obtained by the reaction of nickel(II) nitrate hexa­hydrate with the proton-transfer compound (phenH)_2_(pydc) (phen is 1,10-phenanothroline and pydcH_2_ is pyridine-2,6-dicarboxylic acid) in aqueous solution. Both the cationic and anionic portions of the starting proton-transfer compound are involved in the complexation. The Ni^II^ atom has a distorted octa­hedral geometry and is hexa­coordinated by three O atoms and three N atoms from one phen fragment (as a bidentate ligand), one (pydc)^2−^ unit (as a tridentate ligand) and one water mol­ecule. In the crystal structure, extensive O—H⋯O, O—H⋯N and C—H⋯O hydrogen bonds with *D*⋯*A* distances ranging from 2.573 (2) to 3.385 (2) Å, π–π inter­actions between the phen ring systems [with centroid–centroid distances of 3.4694 (12), 3.4781 (11) and 3.8310 (11) Å] and inter­molecular C—O⋯π inter­actions [C⋯π distances of 3.4812 (17), 3.5784 (16) and 3.5926 (16) Å] connect the various components together.

## Related literature

For proton-transfer compounds see: Aghabozorg *et al.* (2007 ▶); Aghabozorg, Manteghi & Sheshmani (2008 ▶); Aghabozorg, Motyeian *et al.* (2008 ▶); Aharif *et al.* (2007 ▶). For the isostructural Co complex see: Su *et al.* (2005 ▶).
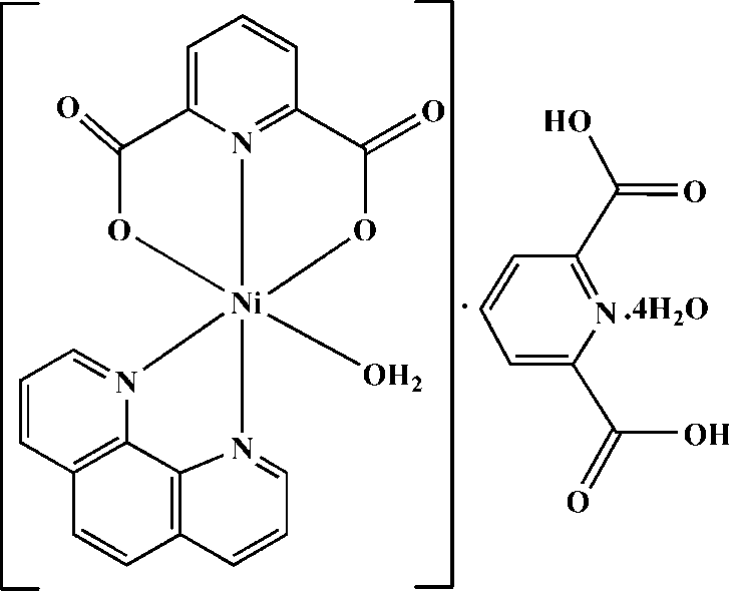


## Experimental

### 

#### Crystal data

[Ni(C_7_H_3_NO_4_)(C_12_H_8_N_2_)(H_2_O)]·C_7_H_5_NO_4_·4H_2_O
*M* *_r_* = 661.22
Triclinic, 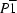

*a* = 9.9454 (7) Å
*b* = 11.3524 (7) Å
*c* = 12.7687 (10) Å
α = 76.527 (2)°
β = 81.252 (2)°
γ = 76.131 (2)°
*V* = 1354.00 (17) Å^3^
*Z* = 2
Mo *K*α radiation
μ = 0.80 mm^−1^
*T* = 100 (2) K
0.41 × 0.32 × 0.26 mm

#### Data collection

Bruker SMART APEXII diffractometer
Absorption correction: multi-scan (*SADABS*; Sheldrick, 1996 ▶) *T*_min_ = 0.736, *T*_max_ = 0.820
14769 measured reflections
6485 independent reflections
5910 reflections with *I* > 2σ(*I*)
*R*_int_ = 0.023

#### Refinement

*R*[*F* ^2^ > 2σ(*F* ^2^)] = 0.034
*wR*(*F* ^2^) = 0.092
*S* = 1.01
6485 reflections
397 parameters
H-atom parameters constrained
Δρ_max_ = 0.47 e Å^−3^
Δρ_min_ = −0.51 e Å^−3^

### 

Data collection: *APEX2* (Bruker, 2007 ▶); cell refinement: *SAINT* (Bruker, 2007 ▶); data reduction: *SAINT*; program(s) used to solve structure: *SHELXS97* (Sheldrick, 2008 ▶); program(s) used to refine structure: *SHELXL97* (Sheldrick, 2008 ▶); molecular graphics: *SHELXTL* (Sheldrick, 2008 ▶) and Mercury (Macrae *et al.*, 2006 ▶); software used to prepare material for publication: *SHELXL97*.

## Supplementary Material

Crystal structure: contains datablocks I, global. DOI: 10.1107/S1600536808039378/om2263sup1.cif

Structure factors: contains datablocks I. DOI: 10.1107/S1600536808039378/om2263Isup2.hkl

Additional supplementary materials:  crystallographic information; 3D view; checkCIF report

## Figures and Tables

**Table:** 

Ni1—N1	1.9790 (15)
Ni1—N2	2.0462 (15)
Ni1—N3	2.0754 (16)
Ni1—O1*W*	2.1023 (13)
Ni1—O1	2.1325 (13)
Ni1—O3	2.1325 (13)

**Table:** 

N1—Ni1—N2	176.22 (6)
N1—Ni1—N3	98.32 (6)
N2—Ni1—N3	80.66 (6)
N1—Ni1—O1*W*	91.23 (6)
N2—Ni1—O1*W*	89.89 (6)
O1—Ni1—O3	155.65 (5)

**Table 2:** Hydrogen-bond geometry (Å, °)

*D*—H⋯*A*	*D*—H	H⋯*A*	*D*⋯*A*	*D*—H⋯*A*
O5—H5*A*⋯O2*W*	0.84	1.96	2.730 (2)	151
O8—H8*A*⋯O4^i^	0.84	1.73	2.573 (2)	177
O1*W*—H1⋯O4^i^	0.85	2.13	2.946 (2)	161
O1*W*—H2⋯O3*W*	0.85	1.95	2.796 (2)	171
O2*W*—H3⋯O2	0.85	2.09	2.900 (2)	159
O2*W*—H4⋯O7	0.85	1.94	2.788 (2)	174
O3*W*—H5⋯O2*W*	0.85	2.09	2.887 (2)	157
O3*W*—H6⋯O4*W*^ii^	0.85	2.37	3.083 (3)	142
O4*W*—H7⋯O1^ii^	0.85	2.03	2.864 (3)	168
O4*W*—H8⋯O5*W*^iii^	0.85	1.93	2.772 (3)	169
O5*W*—H9⋯O2	0.85	2.24	2.878 (3)	131
O2*W*—H4⋯N4	0.85	2.54	2.968 (2)	112
O5—H5*A*⋯N4	0.84	2.18	2.669 (2)	117
C2—H2*A*⋯O6^iv^	0.95	2.28	3.105 (2)	144
C8—H8*B*⋯O1*W*	0.95	2.52	3.070 (2)	117
C12—H12*A*⋯O3^v^	0.95	2.60	3.317 (2)	132
C15—H15*A*⋯O1^vi^	0.95	2.52	3.385 (2)	151

Symmetry codes: (i) 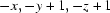; (ii) 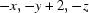; (iii) 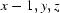; (iv) 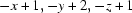; (v) 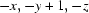; (vi) 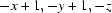.

**Fig. 1.**
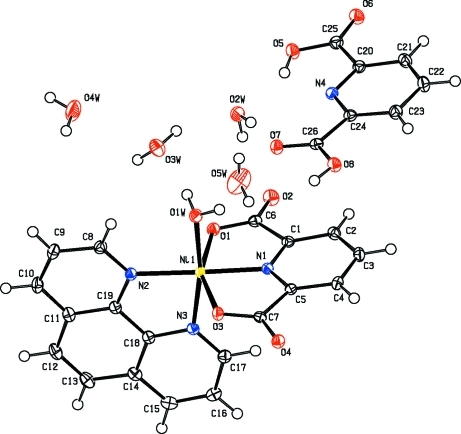
The molecular structure of title compound with displacement ellipsoids drawn at the 50% probability level.

**Fig. 2.**
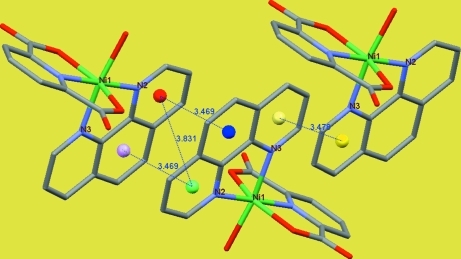
π-π interactions between aromatic rings of 1,10-phenanthroline (phen).

**Fig. 3.**
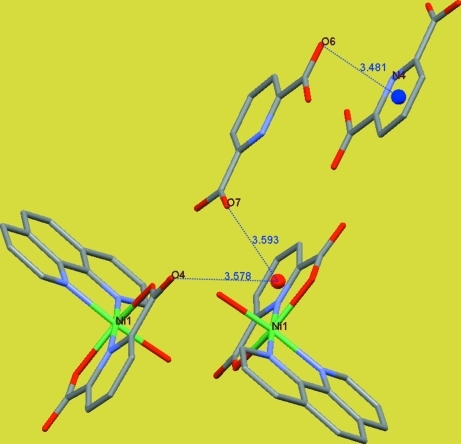
The C–O···π intermolecular interactions between CO of carboxylate groups and aromatic rings.

**Fig. 4.**
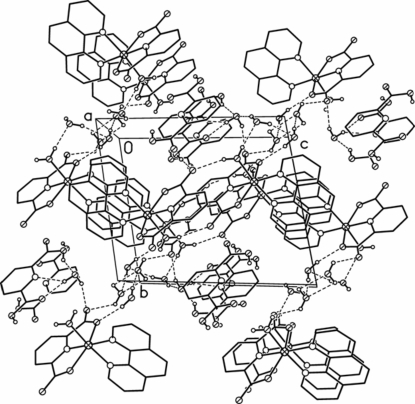
Crystal packing of the title compound along a axis. Hydrogen bonds are shown as dashed lines. The hydrogen atoms that do not take part in hydrogen bonding are not depicted for clarity.

## supplementary crystallographic information

### Comment

Non-covalent interactions including hydrogen bonds are of great importance in stabilizing the structures of different compounds in solid state. The importance of weak hydrogen bonds in the context of crystal engineering, molecular recognition and supramolecular chemistry has been well recognized in recent years. Recently, we have defined a plan to prepare water soluble proton transfer compounds as novel self assembled systems that can function as suitable ligands in the synthesis of metal complexes. In this regard, we have reported cases in which proton transfer from pyridine-2,3-dicarboxylic acid and pyridine-2,6-dicarboxylic acid (pydcH_2_), to piperazine (pipz), 1,10-phenanthroline, (phen) and propane-1,3-diamine (pn), resulted in the formation of self assembled systems. The resulting compounds, with some remaining sites as electron donors, can coordinate to metal ions (Aghabozorg, Daneshvar, *et al.*, 2007; Sharif *et al.*, 2007; Aghabozorg, Motyeian *et al.*, 2008). For more details and related literature see our recent review article (Aghabozorg, Manteghi & Sheshmani (2008).  The title compound is isostructural with a Co complex previously reported  (Su *et al.*, 2005).

The molecular structure of the title compound is presented in Fig. 1. In the [Ni(pydc)(phen)(H_2_O)].(pydcH_2_).4H_2_O compound, both cationic and anionic components of the starting proton transfer compound have been involved in the complexation. The Ni^II^ atom is coordinated by one 1,10-phenanthroline ligand, (phen as bidentate ligand), one pyridine-2,6-dicarboxylate group, ((pydc)^2-^ as tridentate ligand) and one water molecule. The geometry of the resulting NiN_3_O_3_ coordination can be described as distorted octahedral. The angle between the two planes of the (pydc)^2–^ and (phen) ligands is 89.49 (2)°, indicating that these two groups are almost perpendicular to each other.

There are notable π-π interactions between aromatic rings of coordinated (phen) fragments with distances of 3.4781 (11)Å [1 - *x*, 1 - *y*, *-z*], 3.4694 (12)Å [-*x*, 1 - *y*, *-z*] and 3.8310 (11)Å [-*x*, 1 - *y*, *-z*] (Fig. 2). There are also C–O···π intermolecular interactions between CO groups of carboxylate fragments with aromatic rings of pyridine-2,6-dicarboxylate with distances of 3.4812 (17) Å for C25—O6···*Cg*11 (1 - *x*, 2 - *y*, 1 - *z*), 3.5784 (16) Å for C7—O4···*Cg*4 (-*x*, 1 - *y*, 1 - *z*) and 3.5926 (16) Å for C26—O7···*Cg*4 (*x*, *y*, *z*) (Fig. 3). In addition to these interactions there is a wide range of hydrogen bonding of the type O—H···O, O—H···N and C—H···O with D···A ranging from 2.573 (2) to 3.385 (2) Å, (Table 2 and Fig. 4).

### Experimental

The proton transfer ion pair was prepared by a reaction between phen and pydcH_2_. A solution of Ni(NO_3_)_2_.6H_2_O (143 mg, 0.5 mmol) in water (15 ml) was added to an aqueous solution of (phenH)_2_(pydc) (244 mg, 1 mmol) in water (15 ml) in a 1:2 molar ratio. Blue crystals of the title compound suitable for X-ray characterization were obtained after a few days at room temperature.

### Refinement

Hydrogen atoms of hydroxo groups and water molecules were located from the difference Fourier syntheses. The H(C) atoms were placed in geometrically calculated positions. Hydroxo O—H distances were set to 0.84 Å and C—H distances were set to 0.95 Å. Water O—H distances were normalized to 0.85 Å and the hydrogen positions were not further refined. Other hydrogen atom positions were refined with a riding model with the *U*ĩso~(H) parameters equal to 1.2Ueq(O) for hydroxo groups, 1.2Ueq(C) for bonded carbon atoms and to 1.5Ueq(O) for water molecules where *U*~eq~(C) and *U*~eq~(O) are the equivalent isotropic thermal parameters of the atoms to which corresponding H atoms are bonded.

### Crystal data

**Table:**

[Ni(C_7_H_3_NO_4_)(C_12_H_8_N_2_)(H_2_O)]·C_7_H_5_NO_4_·4H_2_O	*Z* = 2
*M**_r_* = 661.22	*F*(000) = 684
Triclinic, *P*1	*D*_x_ = 1.622 Mg m^−^^3^
Hall symbol: -P 1	Mo *K*α radiation, λ = 0.71073 Å
*a* = 9.9454 (7) Å	Cell parameters from 2738 reflections
*b* = 11.3524 (7) Å	θ = 2.6–34.7°
*c* = 12.7687 (10) Å	µ = 0.80 mm^−^^1^
α = 76.527 (2)°	*T* = 100 K
β = 81.252 (2)°	Prism, blue
γ = 76.131 (2)°	0.41 × 0.32 × 0.26 mm
*V* = 1354.00 (17) Å^3^	

### Data collection

**Table:**

Bruker SMART APEXII diffractometer	6485 independent reflections
Radiation source: fine-focus sealed tube	5910 reflections with *I* > 2σ(*I*)
graphite	*R*_int_ = 0.023
ω scans	θ_max_ = 28.0°, θ_min_ = 1.7°
Absorption correction: multi-scan (*SADABS*; Sheldrick, 1996)	*h* = −13→13
*T*_min_ = 0.736, *T*_max_ = 0.820	*k* = −14→14
14769 measured reflections	*l* = −16→16

### Refinement

**Table:**

Refinement on *F*^2^	Primary atom site location: structure-invariant direct methods
Least-squares matrix: full	Secondary atom site location: difference Fourier map
*R*[*F*^2^ > 2σ(*F*^2^)] = 0.034	Hydrogen site location: mixed
*wR*(*F*^2^) = 0.092	H-atom parameters constrained
*S* = 1.01	*w* = 1/[σ^2^(*F*_o_^2^) + (0.04*P*)^2^ + 1.76*P*] where *P* = (*F*_o_^2^ + 2*F*_c_^2^)/3
6485 reflections	(Δ/σ)_max_ < 0.001
397 parameters	Δρ_max_ = 0.47 e Å^−^^3^
0 restraints	Δρ_min_ = −0.51 e Å^−^^3^

### Special details

**Table:**

Geometry. All e.s.d.'s (except the e.s.d. in the dihedral angle between two l.s. planes) are estimated using the full covariance matrix. The cell e.s.d.'s are taken into account individually in the estimation of e.s.d.'s in distances, angles and torsion angles; correlations between e.s.d.'s in cell parameters are only used when they are defined by crystal symmetry. An approximate (isotropic) treatment of cell e.s.d.'s is used for estimating e.s.d.'s involving l.s. planes.
Refinement. Refinement of *F*^2^ against ALL reflections. The weighted *R*-factor *wR* and goodness of fit *S* are based on *F*^2^, conventional *R*-factors *R* are based on *F*, with *F* set to zero for negative *F*^2^. The threshold expression of *F*^2^ > σ(*F*^2^) is used only for calculating *R*-factors(gt) *etc*. and is not relevant to the choice of reflections for refinement. *R*-factors based on *F*^2^ are statistically about twice as large as those based on *F*, and *R*- factors based on ALL data will be even larger.

### Fractional atomic coordinates and isotropic or equivalent isotropic displacement parameters (Å^2^)

**Table:**

	*x*	*y*	*z*	*U*_iso_*/*U*_eq_	
Ni1	0.14557 (2)	0.61408 (2)	0.219863 (18)	0.01073 (7)	
O1	0.27282 (14)	0.74827 (12)	0.17880 (10)	0.0147 (3)	
O2	0.42638 (15)	0.81778 (13)	0.24813 (11)	0.0200 (3)	
O3	0.06221 (13)	0.46491 (12)	0.32080 (10)	0.0135 (3)	
O4	0.07765 (14)	0.33692 (12)	0.48273 (11)	0.0154 (3)	
O5	0.32914 (16)	1.18635 (13)	0.32094 (11)	0.0203 (3)	
H5A	0.2927	1.1262	0.3229	0.024*	
O6	0.43646 (15)	1.24853 (13)	0.43060 (12)	0.0204 (3)	
O7	0.08261 (14)	0.88178 (12)	0.42880 (11)	0.0163 (3)	
O8	0.06159 (14)	0.77645 (12)	0.59989 (11)	0.0171 (3)	
H8A	0.0140	0.7401	0.5742	0.021*	
N1	0.23864 (15)	0.58307 (14)	0.35305 (12)	0.0116 (3)	
N2	0.05900 (16)	0.65260 (14)	0.07697 (12)	0.0129 (3)	
N3	0.29117 (16)	0.49314 (14)	0.13877 (12)	0.0127 (3)	
N4	0.24313 (16)	1.01566 (14)	0.48213 (12)	0.0134 (3)	
C1	0.32172 (18)	0.65734 (16)	0.35902 (14)	0.0121 (3)	
C2	0.37457 (19)	0.65049 (17)	0.45478 (15)	0.0146 (3)	
H2A	0.4327	0.7041	0.4592	0.018*	
C3	0.33980 (19)	0.56227 (18)	0.54497 (15)	0.0155 (4)	
H3A	0.3740	0.5560	0.6120	0.019*	
C4	0.25558 (19)	0.48366 (17)	0.53718 (15)	0.0139 (3)	
H4B	0.2330	0.4222	0.5975	0.017*	
C5	0.20561 (18)	0.49817 (16)	0.43806 (14)	0.0116 (3)	
C6	0.34375 (19)	0.74912 (16)	0.25348 (15)	0.0137 (3)	
C7	0.10751 (18)	0.42734 (16)	0.41213 (14)	0.0118 (3)	
C8	−0.05952 (19)	0.72987 (17)	0.04897 (15)	0.0152 (3)	
H8B	−0.1158	0.7734	0.1008	0.018*	
C9	−0.1049 (2)	0.74989 (18)	−0.05366 (15)	0.0172 (4)	
H9A	−0.1902	0.8060	−0.0704	0.021*	
C10	−0.0251 (2)	0.68771 (18)	−0.12991 (15)	0.0171 (4)	
H10A	−0.0540	0.7010	−0.2001	0.021*	
C11	0.1005 (2)	0.60376 (18)	−0.10257 (15)	0.0154 (4)	
C12	0.1883 (2)	0.53061 (19)	−0.17468 (15)	0.0185 (4)	
H12A	0.1647	0.5407	−0.2461	0.022*	
C13	0.3046 (2)	0.44706 (19)	−0.14207 (16)	0.0186 (4)	
H13A	0.3606	0.3991	−0.1909	0.022*	
C14	0.34446 (19)	0.42989 (17)	−0.03512 (15)	0.0150 (3)	
C15	0.4619 (2)	0.34293 (17)	0.00459 (16)	0.0175 (4)	
H15A	0.5207	0.2914	−0.0402	0.021*	
C16	0.4904 (2)	0.33338 (17)	0.10809 (16)	0.0168 (4)	
H16A	0.5691	0.2750	0.1357	0.020*	
C17	0.40235 (19)	0.41079 (17)	0.17342 (15)	0.0149 (3)	
H17A	0.4235	0.4036	0.2450	0.018*	
C18	0.26186 (19)	0.50255 (16)	0.03579 (14)	0.0130 (3)	
C19	0.13779 (19)	0.58987 (16)	0.00219 (14)	0.0131 (3)	
C20	0.32109 (19)	1.08231 (17)	0.50875 (15)	0.0138 (3)	
C21	0.3586 (2)	1.06598 (18)	0.61277 (16)	0.0175 (4)	
H21A	0.4139	1.1161	0.6285	0.021*	
C22	0.3128 (2)	0.97428 (19)	0.69244 (16)	0.0189 (4)	
H22A	0.3371	0.9596	0.7642	0.023*	
C23	0.2307 (2)	0.90385 (18)	0.66607 (15)	0.0167 (4)	
H23A	0.1978	0.8406	0.7194	0.020*	
C24	0.19803 (18)	0.92821 (16)	0.56006 (15)	0.0131 (3)	
C25	0.36731 (19)	1.17943 (17)	0.41766 (15)	0.0157 (4)	
C26	0.10791 (18)	0.86000 (16)	0.52254 (15)	0.0133 (3)	
O1W	−0.02250 (14)	0.73999 (12)	0.28080 (11)	0.0162 (3)	
H1	−0.0342	0.7339	0.3490	0.024*	
H2	−0.0089	0.8098	0.2446	0.024*	
O2W	0.20162 (16)	1.03365 (13)	0.25314 (11)	0.0206 (3)	
H3	0.2729	0.9812	0.2350	0.031*	
H4	0.1654	0.9911	0.3094	0.031*	
O3W	0.00061 (15)	0.96907 (14)	0.14753 (12)	0.0238 (3)	
H5	0.0484	1.0092	0.1701	0.036*	
H6	0.0279	0.9587	0.0833	0.036*	
O4W	−0.2328 (2)	1.09864 (16)	0.03141 (15)	0.0428 (5)	
H7	−0.2480	1.1528	−0.0263	0.064*	
H8	−0.2752	1.0400	0.0390	0.064*	
O5W	0.5989 (2)	0.92774 (19)	0.06799 (16)	0.0443 (5)	
H9	0.5842	0.8595	0.1070	0.066*	
H10	0.5340	0.9780	0.0964	0.066*	

### Atomic displacement parameters (Å^2^)

**Table:**

	*U*^11^	*U*^22^	*U*^33^	*U*^12^	*U*^13^	*U*^23^
Ni1	0.01194 (12)	0.01138 (12)	0.00901 (11)	−0.00305 (8)	−0.00187 (8)	−0.00123 (8)
O1	0.0164 (6)	0.0140 (6)	0.0136 (6)	−0.0047 (5)	−0.0022 (5)	−0.0008 (5)
O2	0.0198 (7)	0.0206 (7)	0.0217 (7)	−0.0116 (6)	−0.0009 (5)	−0.0020 (6)
O3	0.0145 (6)	0.0154 (6)	0.0113 (6)	−0.0053 (5)	−0.0023 (5)	−0.0013 (5)
O4	0.0178 (6)	0.0146 (6)	0.0139 (6)	−0.0068 (5)	−0.0018 (5)	0.0004 (5)
O5	0.0295 (8)	0.0183 (7)	0.0165 (7)	−0.0131 (6)	−0.0065 (6)	0.0006 (5)
O6	0.0236 (7)	0.0193 (7)	0.0218 (7)	−0.0110 (6)	−0.0049 (6)	−0.0026 (6)
O7	0.0174 (6)	0.0177 (6)	0.0147 (6)	−0.0067 (5)	−0.0028 (5)	−0.0015 (5)
O8	0.0207 (7)	0.0167 (6)	0.0161 (6)	−0.0099 (5)	−0.0029 (5)	−0.0009 (5)
N1	0.0111 (7)	0.0117 (7)	0.0119 (7)	−0.0023 (5)	−0.0011 (5)	−0.0028 (5)
N2	0.0151 (7)	0.0126 (7)	0.0115 (7)	−0.0050 (6)	−0.0021 (6)	−0.0009 (5)
N3	0.0148 (7)	0.0121 (7)	0.0121 (7)	−0.0057 (6)	−0.0012 (6)	−0.0013 (5)
N4	0.0136 (7)	0.0131 (7)	0.0138 (7)	−0.0034 (6)	−0.0020 (6)	−0.0023 (6)
C1	0.0102 (8)	0.0115 (8)	0.0144 (8)	−0.0015 (6)	−0.0009 (6)	−0.0034 (6)
C2	0.0121 (8)	0.0163 (8)	0.0171 (9)	−0.0038 (7)	−0.0020 (7)	−0.0056 (7)
C3	0.0137 (8)	0.0196 (9)	0.0139 (8)	−0.0016 (7)	−0.0043 (6)	−0.0048 (7)
C4	0.0133 (8)	0.0153 (8)	0.0119 (8)	−0.0018 (7)	−0.0024 (6)	−0.0009 (6)
C5	0.0109 (8)	0.0113 (8)	0.0123 (8)	−0.0012 (6)	−0.0015 (6)	−0.0027 (6)
C6	0.0134 (8)	0.0123 (8)	0.0146 (8)	−0.0016 (6)	0.0001 (6)	−0.0032 (6)
C7	0.0117 (8)	0.0119 (8)	0.0121 (8)	−0.0026 (6)	0.0009 (6)	−0.0038 (6)
C8	0.0165 (9)	0.0143 (8)	0.0145 (9)	−0.0040 (7)	−0.0027 (7)	−0.0007 (7)
C9	0.0171 (9)	0.0178 (9)	0.0159 (9)	−0.0050 (7)	−0.0053 (7)	0.0017 (7)
C10	0.0194 (9)	0.0207 (9)	0.0122 (8)	−0.0081 (7)	−0.0046 (7)	0.0010 (7)
C11	0.0167 (9)	0.0184 (9)	0.0126 (8)	−0.0079 (7)	−0.0010 (7)	−0.0021 (7)
C12	0.0218 (9)	0.0244 (10)	0.0123 (8)	−0.0098 (8)	−0.0003 (7)	−0.0055 (7)
C13	0.0194 (9)	0.0244 (10)	0.0148 (9)	−0.0098 (8)	0.0033 (7)	−0.0077 (7)
C14	0.0159 (8)	0.0161 (8)	0.0145 (8)	−0.0075 (7)	0.0014 (7)	−0.0035 (7)
C15	0.0166 (9)	0.0158 (9)	0.0205 (9)	−0.0058 (7)	0.0044 (7)	−0.0060 (7)
C16	0.0140 (8)	0.0146 (8)	0.0202 (9)	−0.0029 (7)	−0.0001 (7)	−0.0016 (7)
C17	0.0157 (8)	0.0141 (8)	0.0148 (8)	−0.0051 (7)	−0.0007 (7)	−0.0012 (7)
C18	0.0149 (8)	0.0134 (8)	0.0117 (8)	−0.0072 (7)	−0.0002 (6)	−0.0009 (6)
C19	0.0149 (8)	0.0136 (8)	0.0116 (8)	−0.0065 (7)	−0.0007 (6)	−0.0012 (6)
C20	0.0125 (8)	0.0136 (8)	0.0153 (8)	−0.0024 (6)	−0.0021 (6)	−0.0028 (7)
C21	0.0177 (9)	0.0189 (9)	0.0190 (9)	−0.0062 (7)	−0.0039 (7)	−0.0060 (7)
C22	0.0218 (9)	0.0226 (9)	0.0135 (9)	−0.0056 (8)	−0.0044 (7)	−0.0038 (7)
C23	0.0188 (9)	0.0167 (9)	0.0146 (9)	−0.0053 (7)	−0.0021 (7)	−0.0012 (7)
C24	0.0119 (8)	0.0120 (8)	0.0151 (8)	−0.0021 (6)	−0.0011 (6)	−0.0030 (6)
C25	0.0157 (8)	0.0144 (8)	0.0172 (9)	−0.0033 (7)	−0.0026 (7)	−0.0028 (7)
C26	0.0121 (8)	0.0113 (8)	0.0163 (8)	−0.0019 (6)	−0.0009 (6)	−0.0031 (6)
O1W	0.0178 (6)	0.0170 (6)	0.0126 (6)	−0.0011 (5)	−0.0008 (5)	−0.0037 (5)
O2W	0.0291 (8)	0.0170 (7)	0.0158 (7)	−0.0069 (6)	−0.0043 (6)	−0.0001 (5)
O3W	0.0238 (7)	0.0283 (8)	0.0233 (8)	−0.0102 (6)	−0.0030 (6)	−0.0081 (6)
O4W	0.0644 (13)	0.0272 (9)	0.0417 (11)	−0.0193 (9)	−0.0339 (10)	0.0116 (8)
O5W	0.0342 (10)	0.0487 (11)	0.0398 (11)	−0.0134 (9)	0.0049 (8)	0.0100 (9)

### Geometric parameters (Å, °)

**Table:**

Ni1—N1	1.9790 (15)	C9—H9A	0.9500
Ni1—N2	2.0462 (15)	C10—C11	1.414 (3)
Ni1—N3	2.0754 (16)	C10—H10A	0.9500
Ni1—O1W	2.1023 (13)	C11—C19	1.406 (3)
Ni1—O1	2.1325 (13)	C11—C12	1.436 (3)
Ni1—O3	2.1325 (13)	C12—C13	1.360 (3)
O1—C6	1.272 (2)	C12—H12A	0.9500
O2—C6	1.246 (2)	C13—C14	1.436 (3)
O3—C7	1.257 (2)	C13—H13A	0.9500
O4—C7	1.260 (2)	C14—C18	1.404 (3)
O5—C25	1.326 (2)	C14—C15	1.411 (3)
O5—H5A	0.8400	C15—C16	1.368 (3)
O6—C25	1.212 (2)	C15—H15A	0.9500
O7—C26	1.217 (2)	C16—C17	1.409 (3)
O8—C26	1.312 (2)	C16—H16A	0.9500
O8—H8A	0.8400	C17—H17A	0.9500
N1—C5	1.331 (2)	C18—C19	1.437 (3)
N1—C1	1.335 (2)	C20—C21	1.393 (3)
N2—C8	1.332 (2)	C20—C25	1.504 (3)
N2—C19	1.362 (2)	C21—C22	1.385 (3)
N3—C17	1.324 (2)	C21—H21A	0.9500
N3—C18	1.364 (2)	C22—C23	1.394 (3)
N4—C20	1.331 (2)	C22—H22A	0.9500
N4—C24	1.339 (2)	C23—C24	1.389 (3)
C1—C2	1.382 (3)	C23—H23A	0.9500
C1—C6	1.522 (2)	C24—C26	1.506 (3)
C2—C3	1.399 (3)	O1W—H1	0.8500
C2—H2A	0.9500	O1W—H2	0.8501
C3—C4	1.390 (3)	O2W—H3	0.8500
C3—H3A	0.9500	O2W—H4	0.8500
C4—C5	1.390 (2)	O3W—H5	0.8501
C4—H4B	0.9500	O3W—H6	0.8501
C5—C7	1.519 (2)	O4W—H7	0.8499
C8—C9	1.402 (3)	O4W—H8	0.8500
C8—H8B	0.9500	O5W—H9	0.8500
C9—C10	1.376 (3)	O5W—H10	0.8500
			
N1—Ni1—N2	176.22 (6)	C9—C10—H10A	120.4
N1—Ni1—N3	98.32 (6)	C11—C10—H10A	120.4
N2—Ni1—N3	80.66 (6)	C19—C11—C10	117.33 (17)
N1—Ni1—O1W	91.23 (6)	C19—C11—C12	119.15 (17)
N2—Ni1—O1W	89.89 (6)	C10—C11—C12	123.49 (17)
N3—Ni1—O1W	170.36 (6)	C13—C12—C11	120.85 (18)
N1—Ni1—O1	78.03 (6)	C13—C12—H12A	119.6
N2—Ni1—O1	98.33 (6)	C11—C12—H12A	119.6
N3—Ni1—O1	91.53 (5)	C12—C13—C14	121.23 (18)
O1W—Ni1—O1	91.76 (5)	C12—C13—H13A	119.4
N1—Ni1—O3	77.62 (6)	C14—C13—H13A	119.4
N2—Ni1—O3	106.01 (5)	C18—C14—C15	117.11 (17)
N3—Ni1—O3	91.48 (5)	C18—C14—C13	118.81 (17)
O1W—Ni1—O3	89.24 (5)	C15—C14—C13	124.07 (18)
O1—Ni1—O3	155.65 (5)	C16—C15—C14	119.53 (17)
C6—O1—Ni1	114.67 (11)	C16—C15—H15A	120.2
C7—O3—Ni1	114.38 (11)	C14—C15—H15A	120.2
C25—O5—H5A	109.5	C15—C16—C17	119.54 (18)
C26—O8—H8A	109.5	C15—C16—H16A	120.2
C5—N1—C1	121.56 (16)	C17—C16—H16A	120.2
C5—N1—Ni1	119.38 (12)	N3—C17—C16	122.47 (17)
C1—N1—Ni1	118.66 (12)	N3—C17—H17A	118.8
C8—N2—C19	117.89 (16)	C16—C17—H17A	118.8
C8—N2—Ni1	128.89 (13)	N3—C18—C14	123.15 (17)
C19—N2—Ni1	113.21 (12)	N3—C18—C19	116.67 (16)
C17—N3—C18	118.21 (16)	C14—C18—C19	120.16 (17)
C17—N3—Ni1	129.43 (13)	N2—C19—C11	123.22 (17)
C18—N3—Ni1	112.35 (12)	N2—C19—C18	116.97 (16)
C20—N4—C24	117.95 (16)	C11—C19—C18	119.77 (17)
N1—C1—C2	121.05 (16)	N4—C20—C21	123.74 (17)
N1—C1—C6	113.02 (15)	N4—C20—C25	115.21 (16)
C2—C1—C6	125.88 (16)	C21—C20—C25	121.05 (17)
C1—C2—C3	117.96 (17)	C22—C21—C20	117.81 (17)
C1—C2—H2A	121.0	C22—C21—H21A	121.1
C3—C2—H2A	121.0	C20—C21—H21A	121.1
C4—C3—C2	120.48 (17)	C21—C22—C23	119.22 (18)
C4—C3—H3A	119.8	C21—C22—H22A	120.4
C2—C3—H3A	119.8	C23—C22—H22A	120.4
C5—C4—C3	117.65 (17)	C24—C23—C22	118.47 (17)
C5—C4—H4B	121.2	C24—C23—H23A	120.8
C3—C4—H4B	121.2	C22—C23—H23A	120.8
N1—C5—C4	121.29 (16)	N4—C24—C23	122.80 (17)
N1—C5—C7	111.82 (15)	N4—C24—C26	113.94 (16)
C4—C5—C7	126.87 (16)	C23—C24—C26	123.26 (16)
O2—C6—O1	126.39 (17)	O6—C25—O5	120.81 (17)
O2—C6—C1	118.29 (16)	O6—C25—C20	122.40 (17)
O1—C6—C1	115.32 (16)	O5—C25—C20	116.79 (16)
O3—C7—O4	125.29 (17)	O7—C26—O8	124.45 (17)
O3—C7—C5	116.47 (15)	O7—C26—C24	121.99 (16)
O4—C7—C5	118.24 (16)	O8—C26—C24	113.56 (16)
N2—C8—C9	122.90 (18)	Ni1—O1W—H1	116.7
N2—C8—H8B	118.6	Ni1—O1W—H2	102.9
C9—C8—H8B	118.6	H1—O1W—H2	114.9
C10—C9—C8	119.48 (18)	H3—O2W—H4	101.8
C10—C9—H9A	120.3	H5—O3W—H6	113.4
C8—C9—H9A	120.3	H7—O4W—H8	113.0
C9—C10—C11	119.17 (17)	H9—O5W—H10	99.9
			
N1—Ni1—O1—C6	−1.09 (12)	N1—C5—C7—O4	173.91 (15)
N2—Ni1—O1—C6	179.94 (12)	C4—C5—C7—O4	−7.9 (3)
N3—Ni1—O1—C6	−99.28 (13)	C19—N2—C8—C9	−0.7 (3)
O1W—Ni1—O1—C6	89.79 (13)	Ni1—N2—C8—C9	178.26 (13)
O3—Ni1—O1—C6	−2.3 (2)	N2—C8—C9—C10	0.1 (3)
N1—Ni1—O3—C7	−0.40 (12)	C8—C9—C10—C11	0.7 (3)
N2—Ni1—O3—C7	178.50 (12)	C9—C10—C11—C19	−0.8 (3)
N3—Ni1—O3—C7	97.79 (13)	C9—C10—C11—C12	177.34 (18)
O1W—Ni1—O3—C7	−91.82 (12)	C19—C11—C12—C13	1.2 (3)
O1—Ni1—O3—C7	0.8 (2)	C10—C11—C12—C13	−176.87 (18)
N3—Ni1—N1—C5	−93.15 (14)	C11—C12—C13—C14	−0.6 (3)
O1W—Ni1—N1—C5	85.50 (13)	C12—C13—C14—C18	−0.9 (3)
O1—Ni1—N1—C5	177.05 (14)	C12—C13—C14—C15	178.47 (18)
O3—Ni1—N1—C5	−3.45 (13)	C18—C14—C15—C16	−0.3 (3)
N3—Ni1—N1—C1	94.01 (13)	C13—C14—C15—C16	−179.67 (18)
O1W—Ni1—N1—C1	−87.35 (13)	C14—C15—C16—C17	−0.2 (3)
O1—Ni1—N1—C1	4.20 (13)	C18—N3—C17—C16	0.0 (3)
O3—Ni1—N1—C1	−176.30 (14)	Ni1—N3—C17—C16	178.89 (13)
N3—Ni1—N2—C8	177.64 (16)	C15—C16—C17—N3	0.3 (3)
O1W—Ni1—N2—C8	−0.40 (16)	C17—N3—C18—C14	−0.5 (3)
O1—Ni1—N2—C8	−92.17 (16)	Ni1—N3—C18—C14	−179.59 (14)
O3—Ni1—N2—C8	88.78 (16)	C17—N3—C18—C19	177.93 (16)
N3—Ni1—N2—C19	−3.34 (12)	Ni1—N3—C18—C19	−1.15 (19)
O1W—Ni1—N2—C19	178.62 (12)	C15—C14—C18—N3	0.7 (3)
O1—Ni1—N2—C19	86.85 (12)	C13—C14—C18—N3	−179.93 (16)
O3—Ni1—N2—C19	−92.20 (12)	C15—C14—C18—C19	−177.73 (16)
N1—Ni1—N3—C17	7.14 (16)	C13—C14—C18—C19	1.7 (3)
N2—Ni1—N3—C17	−176.54 (16)	C8—N2—C19—C11	0.6 (3)
O1—Ni1—N3—C17	85.27 (16)	Ni1—N2—C19—C11	−178.56 (14)
O3—Ni1—N3—C17	−70.56 (16)	C8—N2—C19—C18	−177.09 (16)
N1—Ni1—N3—C18	−173.91 (12)	Ni1—N2—C19—C18	3.77 (19)
N2—Ni1—N3—C18	2.41 (12)	C10—C11—C19—N2	0.2 (3)
O1—Ni1—N3—C18	−95.78 (12)	C12—C11—C19—N2	−178.06 (17)
O3—Ni1—N3—C18	108.39 (12)	C10—C11—C19—C18	177.79 (16)
C5—N1—C1—C2	−1.3 (3)	C12—C11—C19—C18	−0.4 (3)
Ni1—N1—C1—C2	171.37 (13)	N3—C18—C19—N2	−1.7 (2)
C5—N1—C1—C6	−178.88 (15)	C14—C18—C19—N2	176.74 (16)
Ni1—N1—C1—C6	−6.2 (2)	N3—C18—C19—C11	−179.51 (16)
N1—C1—C2—C3	0.8 (3)	C14—C18—C19—C11	−1.0 (3)
C6—C1—C2—C3	178.04 (17)	C24—N4—C20—C21	0.2 (3)
C1—C2—C3—C4	0.6 (3)	C24—N4—C20—C25	−179.58 (16)
C2—C3—C4—C5	−1.4 (3)	N4—C20—C21—C22	0.6 (3)
C1—N1—C5—C4	0.4 (3)	C25—C20—C21—C22	−179.68 (17)
Ni1—N1—C5—C4	−172.21 (13)	C20—C21—C22—C23	−0.7 (3)
C1—N1—C5—C7	178.71 (15)	C21—C22—C23—C24	0.2 (3)
Ni1—N1—C5—C7	6.08 (19)	C20—N4—C24—C23	−0.8 (3)
C3—C4—C5—N1	0.9 (3)	C20—N4—C24—C26	178.62 (16)
C3—C4—C5—C7	−177.09 (17)	C22—C23—C24—N4	0.6 (3)
Ni1—O1—C6—O2	179.10 (15)	C22—C23—C24—C26	−178.76 (17)
Ni1—O1—C6—C1	−1.73 (19)	N4—C20—C25—O6	178.52 (18)
N1—C1—C6—O2	−175.69 (16)	C21—C20—C25—O6	−1.3 (3)
C2—C1—C6—O2	6.9 (3)	N4—C20—C25—O5	−1.7 (2)
N1—C1—C6—O1	5.1 (2)	C21—C20—C25—O5	178.54 (17)
C2—C1—C6—O1	−172.37 (17)	N4—C24—C26—O7	1.8 (3)
Ni1—O3—C7—O4	−176.60 (14)	C23—C24—C26—O7	−178.84 (18)
Ni1—O3—C7—C5	3.63 (19)	N4—C24—C26—O8	−178.54 (15)
N1—C5—C7—O3	−6.3 (2)	C23—C24—C26—O8	0.9 (3)
C4—C5—C7—O3	171.87 (17)		

### Hydrogen-bond geometry (Å, °)

**Table:**

*D*—H···*A*	*D*—H	H···*A*	*D*···*A*	*D*—H···*A*
O5—H5A···O2W	0.84	1.96	2.730 (2)	151
O8—H8A···O4^i^	0.84	1.73	2.573 (2)	177
O1W—H1···O4^i^	0.85	2.13	2.946 (2)	161
O1W—H2···O3W	0.85	1.95	2.796 (2)	171
O2W—H3···O2	0.85	2.09	2.900 (2)	159
O2W—H4···O7	0.85	1.94	2.788 (2)	174
O3W—H5···O2W	0.85	2.09	2.887 (2)	157
O3W—H6···O4W^ii^	0.85	2.37	3.083 (3)	142
O4W—H7···O1^ii^	0.85	2.03	2.864 (3)	168
O4W—H8···O5W^iii^	0.85	1.93	2.772 (3)	169
O5W—H9···O2	0.85	2.24	2.878 (3)	131
O2W—H4···N4	0.85	2.54	2.968 (2)	112
O5—H5A···N4	0.84	2.18	2.669 (2)	117
C2—H2A···O6^iv^	0.95	2.28	3.105 (2)	144
C8—H8B···O1W	0.95	2.52	3.070 (2)	117
C12—H12A···O3^v^	0.95	2.60	3.317 (2)	132
C15—H15A···O1^vi^	0.95	2.52	3.385 (2)	151

Symmetry codes:  (i) −*x*, −*y*+1, −*z*+1; (ii) −*x*, −*y*+2, −*z*; (iii) *x*−1, *y*, *z*; (iv) −*x*+1, −*y*+2, −*z*+1; (v) −*x*, −*y*+1, −*z*; (vi) −*x*+1, −*y*+1, −*z*.
